# Constraint-induced sound therapy for sudden sensorineural hearing loss – behavioral and neurophysiological outcomes

**DOI:** 10.1038/srep03927

**Published:** 2014-01-29

**Authors:** Hidehiko Okamoto, Munehisa Fukushima, Henning Teismann, Lothar Lagemann, Tadashi Kitahara, Hidenori Inohara, Ryusuke Kakigi, Christo Pantev

**Affiliations:** 1 Department of Integrative Physiology, National Institute for Physiological Sciences, Okazaki, Aichi 444-8585, Japan; 2 Institute for Biomagnetism and Biosignalanalysis, University of Muenster, 48149 Muenster, Germany; 3 Department of Otolaryngology, Osaka Rosai Hospital, Sakai, Osaka 591-8025, Japan; 4 Institute for Epidemiology and Social Medicine, University of Muenster, 48149 Muenster, Germany; 5 Department of Otorhinolaryngology Head and Neck Surgery, Osaka University Graduate School of Medicine, Suita, Osaka 565-0871, Japan

## Abstract

Sudden sensorineural hearing loss is characterized by acute, idiopathic hearing deterioration. We report here the development and evaluation of “constraint-induced sound therapy”, which is based on a well-established neuro-rehabilitation approach, and which is characterized by the plugging of the intact ear (“constraint”) and the simultaneous, extensive stimulation of the affected ear with music. The sudden sensorineural hearing loss patients who received the constraint-induced sound therapy in addition to the standard corticosteroid therapy showed significantly better recovery of hearing function compared to those who had only received corticosteroid treatments. Additionally, the brain activity obtained in a subgroup of patients suggested that the constraint-induced sound therapy could have prevented maladaptive auditory cortex reorganization. Constraint-induced sound therapy thus appears to be an effective, practical, and safe treatment option for sudden sensorineural hearing loss.

The US National Institute for Deafness and Communication Disorders defines sudden sensorineural hearing loss (SSHL) as an idiopathic condition characterized by acute hearing loss of at least 30 dB at three or more adjacent frequencies on a standard pure tone audiogram^1^. By definition, SSHL either occurs instantaneously or develops over the course of up to three days. Based on several national surveys^2^,^3^,^4^,^5^, estimates of SSHL incidence rates range from 5–30 cases per 100,000 people per year. A recent study^6^, however, reported an incidence rate as high as 160. Notably, the actual incidence rate is probably even higher, because many SSHL patients do not seek medical treatment.

The likelihood of hearing recovery strongly depends on both the severity of hearing loss at presentation and the time between SSHL incidence and initial audiogram. An 8-year prospective study^2^ of 225 SSHL patients showed that normal or complete recovery occurred in 45% of patients, and a 5-year prospective study^7^ of 166 SSHL patients demonstrated that 65% of patients recovered to functional hearing levels spontaneously and independently of any type of medical treatment. Even though causes and mechanisms have been investigated intensively, knowledge and understanding of SSHL remains to be limited.

In around 10% of SSHL patients^8^ an identifiable cause can be defined, such as Ménière's disease^9^, head trauma, autoimmune inner ear disease^10^,^11^, Cogan's syndrome^12^,^13^, genetic diseases^14^, ototoxic drugs^15^, retrocochlear disorders related to vestibular schwannoma^16^, auditory neuropathy^17^, or stroke^18^. The remaining 90% of cases are often classified as suffering from idiopathic SSHL. While many potential causes of idiopathic SSHL have been proposed, the two most common hypotheses are: 1) circulatory disturbance to the end artery of the cochlea^19^, and 2) viral infection (such as Epstein-Barr^20^, mumps^21^, or varicella-zoster^22^). Based on these hypotheses, several treatment strategies have been proposed and applied, among them vasodilators, antiviral agents, vitamins, and, most commonly, corticosteroids^23^,^24^,^25^,^26^. It is, however, still unknown whether circulatory disturbances^27^,^28^,^29^ and viral infections^30^,^31^,^32^ actually are common causes of SSHL. Moreover, the efficacy of the corticosteroid treatment approach is fiercely disputed, since patients who had received corticosteroids did not recover better than non-treated patients^3^,^33^,^34^,^35^,^36^. Specifically, also a recent randomized triple-blind placebo-controlled clinical trial of 103 SSHL patients^36^ demonstrated that corticosteroids given in customary dosage did not influence hearing recovery. New strategies to treat SSHL are therefore strongly desired.

The occurrence of SSHL changes not only cochlear activity, but also neural activity in the central auditory system. Several neuroimaging studies in humans indicated that SSHL could induce cortical plasticity in the auditory cortex. By means of functional magnetic resonance imaging (fMRI), Scheffler et al.^37^ revealed that monaural sound stimulation of the healthy ear of unilaterally hearing impaired subjects elicited similar neural activity in both hemispheres, whereas monaural stimulation caused neural activity to be strongly lateralized to the contralateral hemisphere in normal-hearing volunteers. In SSHL patients, magnetoencephalography^38^,^39^ and fMRI^40^ studies demonstrated that reorganization of the auditory cortex developed within a few days after the onset of hearing loss. Moreover, the degree of cortical reorganization in the acute SSHL phase correlated negatively with recovery rate from the hearing loss^41^. It has therefore been assumed that the prevention of maladaptive cortical reorganization associated with SSHL may be a promising treatment strategy.

In order to prevent or reduce maladaptive cortical reorganization, we adopted the concept of “constraint-induced therapy”, which is currently used most often in stroke rehabilitation: there, constraint-induced movement therapy urges hemiplegic patients to use their affected limbs and at the same time prohibits their use of the healthy counterpart with physical constraints^42^,^43^,^44^,^45^,^46^. Constraint-induced movement therapy thus prevents or reduces maladaptive cortical reorganization and promotes the recovery of the affected body parts^47^,^48^,^49^,^50^.

In the present study, we plugged (i.e. constrained) the canal of the unaffected ear of acute idiopathic SSHL patients and urged them to actively use the affected ear (Figure 1) by listening to music through a headphone over the affected ear for 6 hours per day. Treatment outcome was evaluated by comparing the pure tone audiograms of two groups of SSHL patients: the control group (N = 31) received only the standard corticosteroid therapy (SCT), while the target group (N = 22) received the constraint-induced sound therapy (CIST) in addition to SCT. Moreover, by means of magnetoencephalography (MEG) we measured brain activity in a subgroup (N = 6) of target patients who agreed to participate in an MEG measurement and for whom we could arrange MEG measurements before the treatment was started. The goal of these MEG measurements was to investigate the degree of maladaptive cortical reorganization before, after, and three months after the SCT + CIST combination therapy.

## Results

No adverse events associated with CIST were observed. The patients in each of the two groups (SCT + CIST vs. SCT) did not differ regarding their age (mean [95% confidence interval (CI) acquired by bootstrap re-sampling tests (iteration = 10,000)]; SCT + CIST: 46.5 [40.3–52.5] years, SCT: 48.9 [44.0–53.5] years) and time between SSHL incidence and initial audiogram (SCT + CIST: 3.09 [2.50–3.68] days, SCT: 3.19 [2.61–3.74] days). We measured the hearing thresholds of all patients before (1^st^ examination) and after (2^nd^ examination) treatment (time interval between 1^st^ and 2^nd^ measurements: SCT + CIST: 9.41 [8.14–10.68] days, SCT: 10.42 [9.29–11.52] days) by means of a pure tone audiometer. A 3^rd^ audiometric examination took place after discharge from hospital (time interval between 2^nd^ and 3^rd^ measurements: SCT + CIST: 63.45 [51.65–76.05] days, SCT: 84.64 [71.11–99.57] days). Two patients from the SCT + CIST group and three patients from the SCT group refused to participate in the 3^rd^ examination. The reason was that all of these five patients had experienced almost complete hearing recovery (mean hearing level difference between ears: 0.6 [−1.0–2.9] dB) and were thus satisfied with their hearing levels at the stage of the 2^nd^ examination. For these patients, we did not expect further hearing improvement. Therefore, in order to avoid bias from systematically missing values, the data of the 2^nd^ examination was used in lieu of the 3^rd^ examination data.

**Figure 2:**
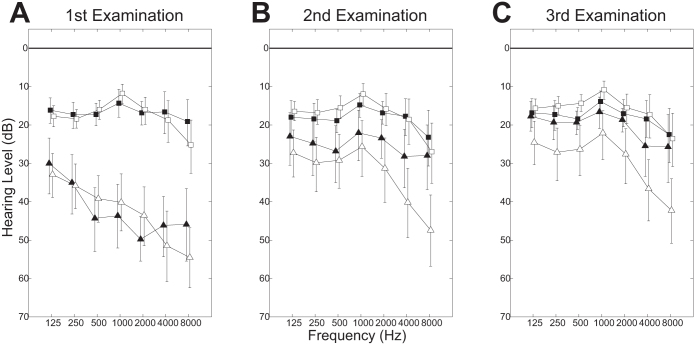
Pure tone audiograms from sudden sensorineural hearing loss patients obtained at the entrance examination (A), at discharge from hospital (B), and at the first outpatient visit after discharge (C). Triangles and squares denote the mean hearing thresholds of the affected and intact ears, respectively. Filled and open symbols indicate the data from the standard corticosteroid therapy + constraint-induced sound therapy (SCT + CIST) and the standard corticosteroid therapy alone (SCT) groups, respectively. The error bars denote 95% confidence intervals.

Figure 2 displays the mean audiograms (range: 125 to 8000 Hz; one octave steps) with 95% CIs of the SCT + CIST and SCT groups in both the affected and intact ears as obtained in the 1^st^ (Figure 2 A), 2^nd^ (Figure 2 B), and 3^rd^ (Figure 2 C) audiometric examinations. Before treatment, at all frequencies in the affected ears, hearing thresholds were similar between groups (mean hearing level (dB) with 95% CI in the SCT + CIST group: 125 Hz 30.2 [23.2–37.7], 250 Hz 35.0 [27.5–43.2], 500 Hz 44.3 [36.4–53.0], 1000 Hz 43.6 [35.5–51.8], 2000 Hz 49.8 [43.9–55.5], 4000 Hz 46.4 [38.4–54.3], 8000 Hz 46.1 [37.0–56.1]; mean hearing level (dB) with 95% CI in the SCT group: 125 Hz 33.1 [27.6–38.9], 250 Hz 35.8 [30.2–41.6], 500 Hz 39.2 [33.1–45.3], 1000 Hz 40.2 [32.9–47.7], 2000 Hz 43.5 [36.0–51.6], 4000 Hz 51.4 [42.6–60.8], 8000 Hz 54.5 [46.6–62.6]). The thresholds of the affected ears had improved in all frequencies in both the SCT + CIST and SCT groups after treatment measured in the 2^nd^ examination (SCT + CIST group: 125 Hz 23.2 [16.8–30.2], 250 Hz 25.0 [18.2–33.2], 500 Hz 27.0 [20.2–35.5], 1000 Hz 22.3 [16.1–29.5], 2000 Hz 23.4 [18.6–28.4], 4000 Hz 28.4 [21.4–36.4], 8000 Hz 28.2 [20.2–36.8], SCT group: 125 Hz 27.3 [21.5–33.4], 250 Hz 30.0 [23.2–37.3], 500 Hz 29.2 [22.4–36.8], 1000 Hz 25.8 [18.7–33.7], 2000 Hz 31.5 [23.4–40.0], 4000 Hz 40.2 [31.3–49.2], 8000 Hz 47.4 [38.2–56.6]) and in the 3^rd^ examination (SCT + CIST group: 125 Hz 17.7 [13.9–21.6], 250 Hz 19.3 [15.0–23.9], 500 Hz 19.3 [16.4–22.5], 1000 Hz 16.6 [12.7–20.7], 2000 Hz 18.6 [15.5–21.8], 4000 Hz 25.7 [18.6–33.4], 8000 Hz 25.9 [17.7–34.8], SCT group: 125 Hz 24.5 [19.0–30.6], 250 Hz 27.3 [20.6–34.4], 500 Hz 26.5 [20.3–33.4], 1000 Hz 22.3 [16.3–28.9], 2000 Hz 27.7 [20.8–35.2], 4000 Hz 36.8 [29.0–45.0], 8000 Hz 42.4 [34.0–50.8]). The mean thresholds of the intact ears did not differ between groups at any of the three examinations (Figure 2). Moreover, the mean thresholds of the intact ears were similar within groups between the three examinations. The observation that the threshold of the intact ear did not change over time in the SCT + CIST group indicates that the plugging of the ear canal did not have an apparent negative effect on the intact ear.

**Figure 3:**
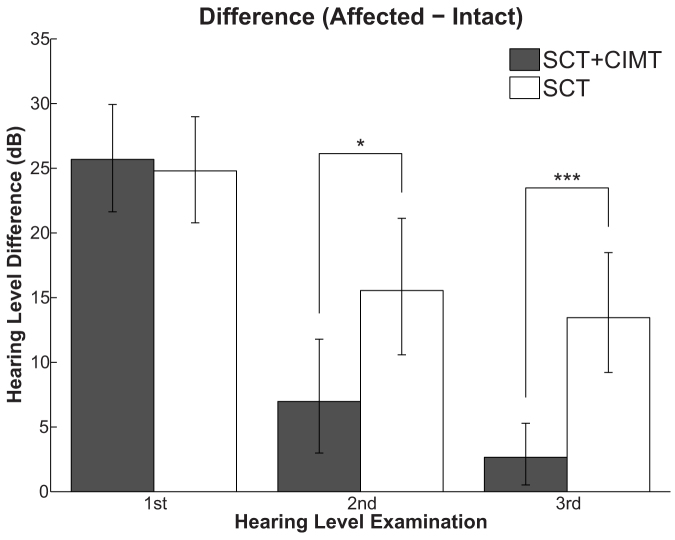
Hearing threshold differences between affected and intact ears averaged across all measured frequencies at the 1^st^ (left: entering hospital), 2^nd^ (centre: leaving hospital), and 3^rd^ (right: first visit as outpatient after discharge) pure tone audiometric examinations. The bars indicate averages of the standard corticosteroid therapy + constraint-induced sound therapy group (SCT + CIST: gray bars) and the standard corticosteroid therapy alone group (SCT: white bars). The error bars denote the 95% confidence intervals. The asterisks indicate significant differences between groups (Bonferroni-adjusted *P* values: *P < 0.05; ***P < 0.001).

The mean threshold differences across all frequencies between the affected and intact ears were similar between the SCT + CIST and SCT groups before treatment (1^st^ examination, Figure 3 A; SCT + CIST: 25.7 [21.7–29.9] dB, SCT: 24.8 [20.7–29.0] dB). However, there were significant differences between the two groups after treatment (2^nd^ examination, Figure 3 B: *U* = 474, *p* < 0.05 (Bonferroni-corrected); SCT + CIST: 7.1 [2.9–11.7] dB, SCT: 15.6 [10.5–21.2] dB) and at the 3^rd^ examination (Figure 3 C: *U* = 547, *p* < 0.001 (Bonferroni-corrected); SCT + CIST: 2.7 [0.5–5.2] dB, SCT: 13.6 [9.3–18.4] dB). The mean threshold differences were significantly smaller in the SCT + CIST group compared to the SCT group in both the 2^nd^ and 3^rd^ examinations.

The 46 patients (SCT + CIST: N = 18; SCT: N = 28) from whom we could obtain informed consent regarding the risks and possible side effects of MRI underwent MRI examinations in order to exclude diseases such as vestibular schwannoma, subdural hematoma, and cerebral infarction. No patient showed pathological remarks. In a follow-up examination more than one year after SSHL onset, no patient reported repetition of hearing loss in the affected or healthy ear; re-occurrence of hearing loss would have been indicative of diseases such as Ménière's disease, vestibular schwannoma, autoimmune inner ear diseases, Cogan's syndrome.

**Figure 4:**
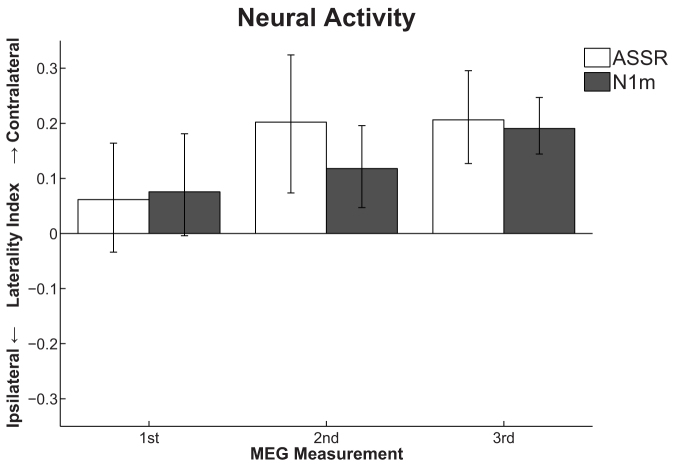
Laterality indices (LIs) of the auditory steady state (ASSR: white bars) and the N1m (gray bars) responses in the standard corticosteroid therapy + constraint-induced sound therapy group (SCT + CIST: N = 6) at the 1^st^ (left: entering hospital), 2^nd^ (centre: leaving hospital), and 3^rd^ (right: three months after discharge) examinations. The error bars denote the 95% confidence intervals. LI = 0 means that the monaural ear stimulation elicited neural activity of equal strength in the contralateral and ipsilateral hemispheres. LI = 1 or LI = −1 means that the neural activity was elicited exclusively in the contralateral or the ipsilateral hemisphere, respectively.

Figure 4 displays the laterality indices (LIs) of auditory steady state (ASSR) and N1m responses for those SCT + CIST group patients who had received MEG measurements (N = 6). The LIs of both ASSR and N1m significantly increased over time (LI of ASSR: F(2,10) = 5.64, *p* < 0.03; LI of N1m: F(2,10) = 6.72, *p* < 0.02). In the 1^st^ examination (prior to SCT + CIST treatment), the LIs of ASSR and N1m were close to 0, meaning that monaural stimulation elicited similar neural activity in the contralateral and ipsilateral hemispheres. However, in the 2^nd^ and 3^rd^ examinations, which took place after SCT + CIST treatment, the LIs of both ASSR and N1m became more positive, indicating that the SSHL patients now showed a dominance of the contralateral hemisphere in response to monaural stimulation, similar to healthy, normal hearing subjects^51^,^52^.

## Discussion

In the target group, which had received the CIST in addition to the SCT, we observed a significantly greater improvement in hearing thresholds compared to the control group, which had received only the SCT. We did not observe any apparent side effects of CIST. Moreover, the neuroimaging results suggest that complementation of SCT with CIST might contribute to the reversal of SSHL-related maladaptive reorganization in primary and non-primary auditory cortex. It therefore appears that the addition of our safe, easy, and cost-effective CIST to the routinely-used corticosteroid treatment is beneficial for SSHL patients.

Crucially, the degree of recovery of hearing thresholds in our control group (SCT) is similar to that reported in an epidemiological study^2^. This study compared the initial audiogram with the best post-SSHL audiogram to give the following criteria for describing hearing recovery: *normal* = return to ≤25 dB hearing level in all tested frequencies; *complete* = return to level of pre-SSHL audiogram (if available), or return to a level within 10 dB of the non-affected ear; *partial* = improvement in mean pure tone audiogram of ≥10 dB; *no recovery* = improvement in pure tone audiogram of ≤9 dB; *worse* = deterioration of mean pure tone audiogram of ≥5 dB. The results showed that 50% of the patients with *intermediate severity of initial hearing loss* (i.e. 35–54 dB hearing level in the affected ear) showed *normal* and *complete* recovery, 20% showed *partial* recovery, and 30% showed *no change*. In our study, the mean hearing levels of the SCT + CIST (42.2 dB) and SCT (42.5 dB) groups after SSHL occurrence were equivalent to the *intermediate severity of initial hearing loss* category (Figure 2 A). We found that, in accordance with the criteria used in the previous study^2^, 58% of the patients in the SCT group showed *normal* and *complete* recovery (18/31), 19% showed *partial* recovery (6/31), 19% showed *no* change (6/31), and in 3% there was deterioration (1/31). Conversely, 86% of the patients in the SCT + CIST group showed *normal* and *complete* recovery (19/22), 14% showed *partial* recovery (3/22), and 0% showed *no* change or deterioration (0/22). The significant difference between the SCT + CIST and SCT groups observed here can therefore not be attributed to the low recovery rate in the SCT group, but reflects the good recovery rate in the SCT + CIST group. Our results indicate that the sound stimulation of the affected ear and the temporary artificial hearing loss induced in the intact ear were beneficial for the recovery of hearing thresholds in the affected ear.

It has been clearly demonstrated that unilateral hearing loss can lead to auditory cortex reorganization^38^,^53^,^54^. For instance, cortical neurons, which were originally activated by the affected cochlea, lose their responsiveness to the acoustic stimulation of the affected ear and instead become responsive to stimulation of the intact ear. The ability of the human cortex to reorganize itself has a 'light' and a 'dark' side^55^. Positive implications of cortical reorganization in the auditory system include training-induced enlargements of cortical areas corresponding to the preeminent auditory performance of musicians^56^, and improved low frequency discrimination abilities of subjects with high frequency hearing loss^57^. On the other hand, there are several disorders which seem to originate from maladaptive cortical reorganization, e.g. focal hand dystonia^58^, phantom limb pain^59^, or tinnitus^60^. Li et al.^41^ demonstrated that a high degree of reorganization was associated with poor recovery from hearing loss in SSHL patients. It is possible that the action of cortical reorganization, in the form of neural recruitment induced by the deprivation of neural activity from the affected ear to the auditory cortex, may compromise hearing recovery in this situation. As a consequence of the SSHL, the auditory neurons that originally corresponded to the affected ear may lose their afferent neural input and may establish new neural connections originating from the intact cochlea. In a normal hearing subject, the cochlea projects afferent neural inputs mainly to the contralateral auditory cortex; therefore, SSHL-related cortical reorganization would primarily occur in the auditory cortex contralateral to the affected ear. The results of the present MEG measurements also support this hypothesis; the amplitude of cortical activity elicited by monaural sound stimulation was similar between the contralateral and ipsilateral hemispheres prior to the SCT + CIST treatment (Figure 4). This kind of cortical reorganization could be maladaptive, because the newly-established connections between the auditory cortex and the intact cochlea would lead to enhanced afferent stimulation of the cortical structure, against which the lower firing rate originating from the affected cochlea could not compete. This more compelling stimulation from the unaffected cochlea would induce more efferent neural inputs from the auditory cortex to the unaffected ear. Over time, the neural signals from the recovering affected ear would lose their salience and would become ignored. As a consequence, maladaptive cortical reorganization would prevent the reestablishment of the affected ear as the primary input source to the contralateral auditory cortex.

Behavioral training which intends to reverse maladaptive cortical reorganization is effective in alleviating symptoms such as focal hand dystonia^61^, phantom limb pain^62^, and tinnitus^63^, and the present results suggest that the prevention of maladaptive auditory cortical reorganization by means of CIST could be an effective, safe, and inexpensive treatment approach for SSHL. In the present study, the SSHL patients of the SCT + CIST group wore an ear plug in the intact ear, while the affected ear was extensively stimulated with music, leading to increased neural activity corresponding to the affected cochlea and reduced neural activity corresponding to the intact cochlea. The present MEG results (Figure 4) demonstrated that the neural activity elicited by monaural sound stimulation was dominant in the contralateral hemisphere after SCT + CIST treatment. This was the case for both primary auditory cortex (ASSR) and auditory belt areas (N1m). Such contralateral dominance is also found in healthy, normal hearing subjects. At the 3^rd^ examination, the LI of the N1m response was about 0.2, a value almost equal to the LI found in healthy controls in a previous study^41^. Therefore, SCT + CIST would both promote neural reconnection between the auditory cortex and the affected cochlea and reverse maladaptive cortical reorganization, which was caused by imbalanced neural activities from the affected and intact cochleae. However, it is also plausible to assume that the SCT and/or natural hearing recovery might have contributed to the improvements in contralateral-side dominances of the ASSR and N1m responses obtained in the present study. At the moment, we cannot precisely estimate the contribution that CIST made to the reversion of the maladaptive cortical reorganization. In an animal study^64^, cats were exposed to traumatizing noise and, immediately thereafter, were stimulated with moderate level sounds for a few weeks (“enriched acoustic environment”). The results showed that the cats that were housed in the enriched acoustic environment had much lower levels of hearing loss and better-preserved tonotopic maps in the primary auditory cortex than cats that were housed in a quiet environment after the traumatizing noise exposure. The authors concluded that the enriched acoustic environment had minimized auditory cortical reorganization. Several studies support the efficacy of sound therapy in humans with hearing loss^65^,^66^,^67^. In the present study, we used classical music as an enriched acoustic environment. We cannot conclude what kind of sound would be best for CIST; however, listening to music is much less distressing than listening to pure tones or noise, and the enjoyment of a stimulus is an important factor in the initiation of cortical reorganization^68^. The use of enjoyable music in our study as an enriched acoustic environment for the affected ear might result in the improved recovery of hearing function, as observed in the SCT + CIST compared to the SCT group.

In the present study, the patients in the SCT + CIST group individually adjusted the spectral properties of the training music via equalizers in order to make it sound as similar as possible to the sound before SSHL emergence. This adjustment resulted in an increase of sound energy in frequency regions affected by hearing loss as well as a decrease of sound energy at unaffected frequencies. Previous studies reported that partial deprivation of neural input from the cochlea to the auditory cortex leads to both the expansion of neural groups corresponding to non-deprived frequencies and the shrinkage of neural groups corresponding to deprived frequencies^69^,^70^. Despite the significant hearing loss caused by SSHL, we assume that the auditory cortex neural activity elicited by the equalized music (i.e., following individuals' adjustment of the frequency spectrum as described above) would be similar to that elicited by normal music via an intact ear. The spectrally balanced neural activity patterns in the auditory cortex may prevent long-term reorganization induced by the spectrally imbalanced afferent inputs consequent to SSHL.

Several studies have reported that sound stimulation dilates blood vessels and increases red blood cell velocity in the cochlea^71^,^72^,^73^,^74^. Improving the microcirculation of the cochlea could be effective in limiting noise-induced hearing loss^75^. In the present study, the exposure to music, which has complex spectral and temporal properties, might have effectively and specifically increased the blood flow in the affected cochlea, and therefore may have dissolved the oxygen deficiency, which is a potential SSHL cause. Even if there was no hypoxia in the affected cochlea, by supplying oxygen and substances necessary for restoration and by removing toxic substances, the increased blood flow may support the recovery of damaged cochlear tissues. Moreover, activating the functional inner hair cells in the affected ear by music may have promoted the release of neurotrophins, which are necessary for the survival of the auditory nerve fibers, and may have facilitated the repair of damaged auditory nerve fibers^76^,^77^,^78^. Electrical stimulation in the cochlea of deafened guinea-pigs has also been shown to promote survival of spiral ganglion neurons^79^,^80^,^81^, and the combination of electric stimulation and neurotrophin infusion appears to further improve and maintain hearing recovery^82^,^83^. The music stimulation applied in the present study may also have facilitated both the neurotrophin release and the auditory nerve fiber activation, leading to better hearing recovery in the SCT + CIST group. The functional recovery of the cochlea and the auditory nerve fibers of the affected side may be able to compete against the newly establishing neural connections between the auditory cortex and the intact cochlea. Thereby CIST may affect both the peripheral and the central structures, and most importantly the interactions between them. Without the peripheral improvement, afferent firing would still be reduced, and the central structure would always seek supplementary stimulation from the intact cochlea. Without the central improvement (i.e. undoing the maladaptive reorganization), the improved peripheral firing would still be ignored and would not have its original destination in the cortex anymore. CIST would contribute to breaking through this vicious cycle of peripheral damage and maladaptive cortical reorganization.

SSHL patients who had interaural differences of greater than 50 dB mean hearing level across 500, 1000, and 2000 Hz were excluded from participation in the present study. The reason for this is that such patients may have been able to hear both the music and environmental sounds in the affected ear despite the presence of the plug in the canal of the intact ear. Due to this so-called “cross-hearing^84^”, such patients might mainly use the intact ear to listen to the training music and to environmental sounds, and this would be in direct conflict with the aim of CIST, which is to enforce the use of the affected ear. Nonetheless, CIST might still be effective because of the enriched acoustic environment effect even when SSHL patients make use of cross-hearing. Further studies are needed to investigate the efficacy of CIST in SSHL patients with severe and profound hearing losses.

Even though potential corticosteroid effects are still under debate, all patients in the present study received the SCT, which is currently the gold standard treatment. We cannot therefore conclude beyond doubt from our results that the superior improvement of the SCT + CIST group was solely caused by the CIST. It is possible that the CIST strengthened the corticosteroid effect, or that the corticosteroids might have enhanced the CIST effect, or both. Moreover, in the present study the participants decided by themselves whether they received CIST or not, and thus the results could theoretically be biased by motivational differences between groups. We are planning to conduct a randomized controlled multicenter trial by means of which the effects of isolated CIST will be compared to SCT effects. Compared to the SCT, which can induce severe and potentially lethal side effects such as infections, diabetes mellitus, and hypertension, CIST might constitute an inexpensive and safe alternative for SSHL treatment.

## Methods

### Subject

54 SSHL patients matching the following criteria were included into the study: i) days since SSHL onset ≤ 5, ii) mean hearing threshold difference across 500, 1000, and 2000 Hz between ears ≤ 50 dB, iii) age ≥ 20 and ≤70 years, iv) unilateral idiopathic hearing loss according to the criteria established by the Sudden Deafness Research Committee of the Ministry of Health and Welfare in Japan (1973), v) no previous self or family history of SSHL, and vi) no neurological or psychiatric complications. These inclusion criteria were used in order to minimize potential complications of interpretation. On admission, eligible patients were asked whether they wished to receive the CIST in addition to the SCT. Those patients who agreed were assigned to the SCT + CIST group; the remaining patients received merely the SCT and constituted a control group.

One patient from the SCT + CIST group dropped out during the first day of treatment due to underestimation of the effort required by participation. In the aftermath, he or she continued to receive the SCT, but his or her data was excluded from subsequent analyses. In total, 53 SSHL patients (SCT + CIST: N = 22, 13 females, 9 males; SCT: N = 31; 14 females, 17 males) were included into the data evaluation. Six SSHL patients (SCT + CIST: 5 females 1 male, 40.8 [34.3–47.0] years) received MEG measurements. All patients were fully informed about the execution and goals of the study and gave written informed consent in accordance with procedures approved by the Ethics Committee of the Medical Faculty, University of Muenster, the Ethics Committee of the Osaka University Hospital, and by the Ethics Committee of the Osaka Rosai Hospital. The study was performed in accordance with the Declaration of Helsinki.

### Study description

During the 1^st^ entrance examination, hearing threshold levels (air and bone conduction) were measured using a step size of 5 dB in accordance with the modified Hughson-Westlake procedure^85^, by means of a pure tone audiometer (AA-78, RION Co., Ltd, Tokyo, Japan, or Orbiter 922DH, GN Otometrics Corporation, Taastrup, Denmark). Thereafter, all patients were admitted to hospital and received a course of corticosteroids tapered over a period of 5 to 14 days (e.g., starting from 3 mg of betamethasone daily for 3 days, followed by 2 mg daily for 3 days, 1 mg daily for 2 days, and 0.5 mg daily for 2 days). The outer canal of the unaffected ear of patients in the SCT + CIST group was plugged all day-long by an ear mold (EM-59, RION Co. Ltd, Tokyo, Japan) for the entire period of their hospitalization. During hospitalization, the SCT + CIST patients listened to classical music for 6 hours per day with the affected ear using a portable music player, equalizer (Equalizer GE-7, Roland Corporation, Hamamatsu, Japan), headphone amplifier (Headphone Amplifier E11, FiiO Electronics Technology Co. Ltd, Guangzhou, China) and closed-type headphone (HD280pro, SENNHEISER electronic GmbH & Co. KG, Hannover, Germany). Patients were instructed to adjust the sound level and equalizer settings by themselves in such a way that the music sounded as similar as possible to the sound before SSHL emergence. This adjustment led to an increase in sound power in the range of the affected frequencies. The hearing threshold levels of the affected ear were measured every two days in order to monitor recovery and to adjust the taper of corticosteroids. When the patients were discharged from hospital (2^nd^ measurement) and when they visited us as outpatients 1 to 6 months after discharge (3^rd^ measurement), hearing thresholds of both the affected and intact ears were measured.

### Brain activity measurements

Auditory evoked neural responses were measured by MEG in a magnetically shielded, silent room. Since MEG systems were not installed in all facilities and since it was difficult to perform unscheduled MEG measurement before starting the SSHL treatment, we could obtain MEG measurements only from six patients of the target (SCT + CIST) group. These patients participated in three MEG sessions (before SCT + CIST treatment, at the end of hospitalization, and 3 months after discharge). During the MEG measurements, we used a monaurally presented test stimulus, which was randomly delivered to the affected or the unaffected (healthy) ear. The carrier frequency of the test stimulus corresponded to a patient's worst hearing frequency between 125 and 4000 Hz in the affected ear. The test stimulus had duration of 1 s and was fully amplitude-modulated with a modulation rate of 40 Hz. The utilization of amplitude-modulated sounds enabled us to investigate N1m^86^ (a major deflection of the auditory evoked response originating from auditory belt area) and ASSR^87^ (generated in primary auditory cortex) responses simultaneously^88^. Before each MEG session, the hearing threshold for the test stimulus was determined for the unaffected (healthy) ear. During the actual measurements, the test stimulus was presented with intensity of 45 dB above individual sensation threshold in the unaffected (healthy) ear; a loudness (not intensity) matched test stimulus was presented in the affected ear. The silent interval between two successive test stimuli was randomized between 1 and 2 s. In order to keep participants in a stable alert state, they were instructed to ignore the sound stimuli and to watch a silent movie of their choice during the MEG recordings.

To increase the signal to noise ratio of the evoked responses, we calculated the grand averaged auditory evoked fields elicited by all test sounds across affected and unaffected ears for each MEG session. The obtained ASSR and N1m responses demonstrated clear dipolar patterns. Thus, we used an equivalent current dipole model for source analysis. For the ASSR analysis, the source location and orientation in each hemisphere was estimated to the averaged magnetic field data (band-pass filtered between 32 and 48 Hz) within the 0.7 s time interval starting 0.3 s after test stimulus onset until the end of the stimulus duration. Thereafter, we used the mean source locations and orientations of three MEG sessions as a spatial filter and obtained the neural activity in the left and right hemispheres in each MEG session^89^. In order to investigate the degree of cortical reorganization, we calculated the laterality indices (LIs) of the neural activity in the ipsilateral and contralateral hemispheres. LI was calculated as follows: (A + B − C − D)/(A + B + C + D), A = source strength elicited by the affected ear stimulation in the contralateral hemisphere, B = source strength elicited by the healthy ear stimulation in the contralateral hemisphere, C = source strength elicited by the affected ear stimulation in the ipsilateral hemisphere, D = source strength elicited by the healthy ear stimulation in the ipsilateral hemisphere. For the N1m analysis, the grand-averaged magnetic fields were 30 Hz low-pass filtered and baseline-corrected based on the 0.3 s pre-stimulus silent interval. A 0.01 s time window around the N1m peak was used for dipole source estimation. We estimated the source location and orientation of the equivalent current dipole in each hemisphere and calculated the LI in a manner similar to the ASSR analysis.

### Statistics

Assuming that the audiogram of the intact ear was similar to the pre-SSHL audiogram of the affected ear, we calculated the mean hearing threshold differences across all frequencies between ears in order to estimate the degree of hearing recovery under treatment. These differences were statistically compared between the two groups (SCT + CIST and SCT) at the 2^nd^ and 3^rd^ audiometric examinations. Given that the data was not normally distributed, Mann-Whitney U tests were calculated. The Bonferroni multiple comparison correction was used to control the family-wise error rate. All statistical analyses were performed using SPSS software (PASW Statistics 18, IBM Corporation, Armonk, NY). The LIs of ASSR and N1m responses were evaluated separately via repeated-measures analyses of variance (ANOVA) using TIME as factor (before SCT + CIST, after SCT + CIST, three months after SCT + CIST). Mauchly's test showed that the sphericity assumption was not violated.

## Author Contributions

H.O. conceived of the study and designed the experimental setup. H.O., M.F., H.T., L.L. and T.K. collected the data. H.O., M.F., H.T. and L.L. performed the data & statistical analyses. H.O. and H.T. wrote the article. H.I., R.K. and C.P. edited the article. All authors have approved the final version of the manuscript.

## Figures and Tables

**Figure 1:**
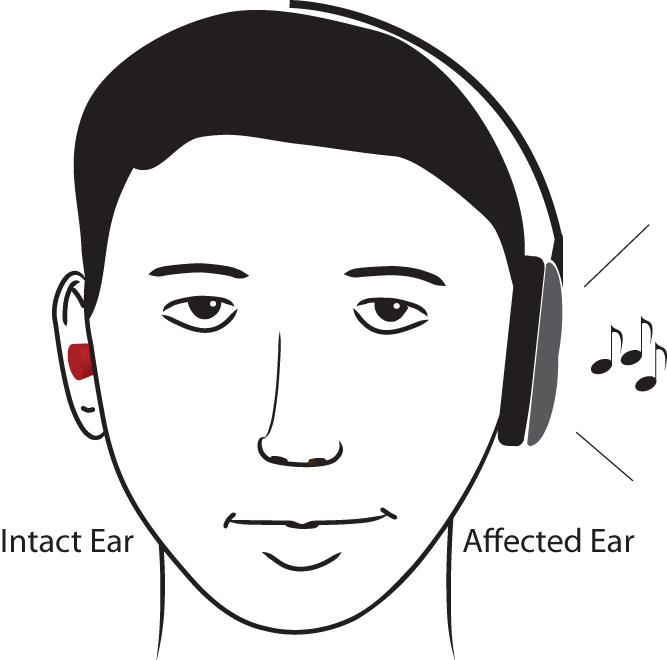
Schematic illustration of the constraint-induced sound therapy (CIST). The canal of the intact ear was plugged. Music was only presented to the affected ear; the other channel of the headphone was silent. (Drawing courtesy of Lothar Lagemann.).
